# Patient-Derived Induced Pluripotent Stem Cell Models for Phenotypic Screening in the Neuronal Ceroid Lipofuscinoses

**DOI:** 10.3390/molecules26206235

**Published:** 2021-10-15

**Authors:** Ahmed Morsy, Angelica V. Carmona, Paul C. Trippier

**Affiliations:** 1 Department of Pharmaceutical Sciences, College of Pharmacy, University of Nebraska Medical Center, Omaha, NE 68106, USA; ahmed.morsy@unmc.edu (A.M.); angelica.carmona@unmc.edu (A.V.C.); 2 Fred & Pamela Buffett Cancer Center, University of Nebraska Medical Center, Omaha, NE 68106, USA; 3 UNMC Center for Drug Discovery, University of Nebraska Medical Center, Omaha, NE 68106, USA

**Keywords:** neuronal ceroid lipofuscinosis, batten disease, induced pluripotent stem cells, screening, model systems, drug discovery

## Abstract

Batten disease or neuronal ceroid lipofuscinosis (NCL) is a group of rare, fatal, inherited neurodegenerative lysosomal storage disorders. Numerous genes (CLN1–CLN8, CLN10–CLN14) were identified in which mutations can lead to NCL; however, the underlying pathophysiology remains elusive. Despite this, the NCLs share some of the same features and symptoms but vary in respect to severity and onset of symptoms by age. Some common symptoms include the progressive loss of vision, mental and motor deterioration, epileptic seizures, premature death, and in the rare adult-onset, dementia. Currently, all forms of NCL are fatal, and no curative treatments are available. Induced pluripotent stem cells (iPSCs) can differentiate into any cell type of the human body. Cells reprogrammed from a patient have the advantage of acquiring disease pathogenesis along with recapitulation of disease-associated phenotypes. They serve as practical model systems to shed new light on disease mechanisms and provide a phenotypic screening platform to enable drug discovery. Herein, we provide an overview of available iPSC models for a number of different NCLs. More specifically, we highlight findings in these models that may spur target identification and drug development.

## 1. Introduction

Batten disease, also known as neuronal ceroid lipofuscinosis (NCL), is a group of rare pediatric neurodegenerative diseases estimated to affect 1 in 12,500 people [1]. The rarity of incidence and scarcity of disease models have limited comprehensive understanding of the pathological factors that lead to disease progression. Batten disease is classified into five primary types:

(A) Congenital NCL, where babies are born with microcephaly as the disease begins in utero [2]. 

(B) Infantile NCL, where symptoms such as seizures and the loss of motor function appear between the ages of 6 and 18 months with the loss of psychomotor skills, including speech. The child presents with signs of regression accompanied by the onset of epilepsy and a gradual loss of vision, hyperexcitability, restlessness, and poor sleep. After the age of 15 to 20 months, the acceleration of symptoms occurs, leading to microcephaly, truncal ataxia, dystonic features, choreoathetosis, and myoclonic jerks. By the age of 24 months, children become blind and lose all cognitive and active motor skills and usually die between the ages of 9 and 13 [3]. 

(C) Late infantile NCL, where symptoms such as developmental delay, ataxia, and seizures appear between the ages of 2 and 4 years old and progress rapidly to loss of motor, cognitive and language functions, ultimately becoming behaviorally abnormal and demented [3]. 

(D) Juvenile NCL (JNCL), the most common type of Batten disease with symptoms occurring between 5 and 10 years old and is commonly associated with the loss of vision and seizures. Further symptoms include learning difficulties, motor disturbances, including extrapyramidal and less prominent pyramidal involvement (rigidity, bradykinesia, slow steps with flexion in hips and knees, and shuffling gait), which appear around puberty and progressively lead to the loss of independent mobility. Affected children usually die during their third decade [3]. 

(E) Adult NCL, where symptoms are less severe and progress more slowly. The clinical picture of this type is characterized by generalized tonic seizures, myoclonus, and prominent dementia. Associated features include speech problems, cerebellar dysfunction, and parkinsonism [4,5]. 

The NCL disorders are genetic conditions primarily inherited in an autosomal recessive manner except in the adult form, where it is autosomal dominant. Associated genes that cause the NCL encode a number of proteins that include lysosomal luminal proteins, intracellular transmembrane proteins, plasma membrane proteins, and cytoplasmic proteins (Table 1) [6]. Because of the different gene mutations, signs and symptoms range in severity and progress at different rates. However, the main characteristics seen in all types of NCLs are nerve cell lysosomal accumulation of auto-fluorescent, electron-dense material, containing subunit c of mitochondrial ATP-synthase and/or sphingolipid activator proteins A and D. Protein mutations in NCL diseases lead to the accumulation of storage material in cells of the eye [7], brain [8], skin [9], muscle [10], and others, ultimately resulting in cell death. Accumulation leads to several mechanisms of neuronal cell death that include accelerated apoptosis and autophagic death, [11] as well as secondary destructive inflammation [12]. An established pathogenetic pathway for this group of diseases remains to be determined despite efforts to develop new biological tools that can further disease understanding and provide biomarkers to monitor disease progression and therapeutic efficacy.

## 2. Approved Therapies and Ongoing Drug Discovery Efforts for the NCLs

With the limited knowledge of NCL pathology, treatment strategies, classified into enzyme therapy, gene therapy and small molecules, are focused on a variety of approaches which have resulted in two drug approvals. In 2017, the U.S. Food and Drug Administration (FDA) approved the enzyme replacement therapy, Brineura, to treat CLN2 disease. Brineura is a recombinant human TPP1 (rhTPP1) proenzyme activated in the acidic environment of the lysosome [34]. The administration of rhTPP1 is performed through catheters implanted in the lateral ventricle (intracerebroventricular) and delivered every other week [35]. Treatment with the rhTPP1 in CLN2 disease patients has shown a significant delay of motor, language and visual decline and substantial reduction in cortical volume loss [34]. In vitro, the mannose-6-phosphate post-translational modification of rhTPP1 enhanced its endocytosis, hence its function towards the restoration of TPP1 activity resulting in reduced storage material accumulation in fibroblasts [36]. 

Another treatment approach used for the NCLs is an adeno-associated virus (AAV)-mediated gene therapy. This treatment strategy was shown to be safe and effective in several clinical trials for lysosomal storage disorders. For more detailed information, this topic was reviewed by Sawamoto et al. in 2018 [37]. The reintroduction of the lysosomal enzyme via gene therapy has demonstrated the rescue of enzyme activity. The intracranial injections of AAV encoding human PPT1 (hPPT1) increased enzyme activity and rescued phenotypic features of Batten disease in a mouse model of CLN1 disease (*Ppt1^−/−^*) [38]. Moreover, the improvement of motor functions and learning behaviors increased with multiple injections in a dose-response manner. In a canine model of CLN2 disease, the intraventricular delivery of AAV encoding canine TPP1 (caTPP1) into the circulating CSF led to widespread transduction of AAV-caTPP1 to the ependymal lining of the third and fourth ventricles and the secretion and increase of the levels of caTPP1 in the cortex and cerebellum [39]. This increase has led to delayed onset of Batten disease symptoms, reduced glial activation, rescued behavioral phenotypes, and increased longevity [39]. In another study, driving the expression of AAV9–hCLN3 vector by one of two promoters, a *Mecp2* promoter or a chicken β-actin (CB) promoter, resulted in three-fold to eight-fold increase in *CLN3* expression when delivered intravenously into *CLN3*^Δ*ex7/8*^ mice. Moreover, this resulted in correcting multiple disease pathologies, including motor coordination, reduced astrocytes, microglial activation, and lysosomal pathology [40,41]. In a phase I/IIa clinical trial (NCT03770572), the use of an AAV9 capsid carrying the AAV2 gene cassette with hCLN3 injected intrathecally into the subarachnoid space of the lumbar spine of patients reported widespread CNS expression and a reduction in pathological hallmarks. Similarly, the administration of ovine CLN5 encoded in AAV9 vectors in a sheep model of CLN5 disease showed a substantial delay in brain atrophy and visual decline [42]. These data supported the benefits of CLN5 gene therapy, and the FDA has granted orphan drug approval to *Neurogene*’s gene therapy towards CLN5 disease [43]. In a CLN6*^nclf^* mouse model, a single intracerebroventricular injection of scAAV9.CB.hCLN6 and intrathecal injection in non-human primates resulted in the increased expression of CLN6 in the CNS, including in the eye and optic nerve. The increase of CLN6 led to a significant reduction in lysosomal storage materials and enhanced autophagy [19]. 

**Figure 1:**
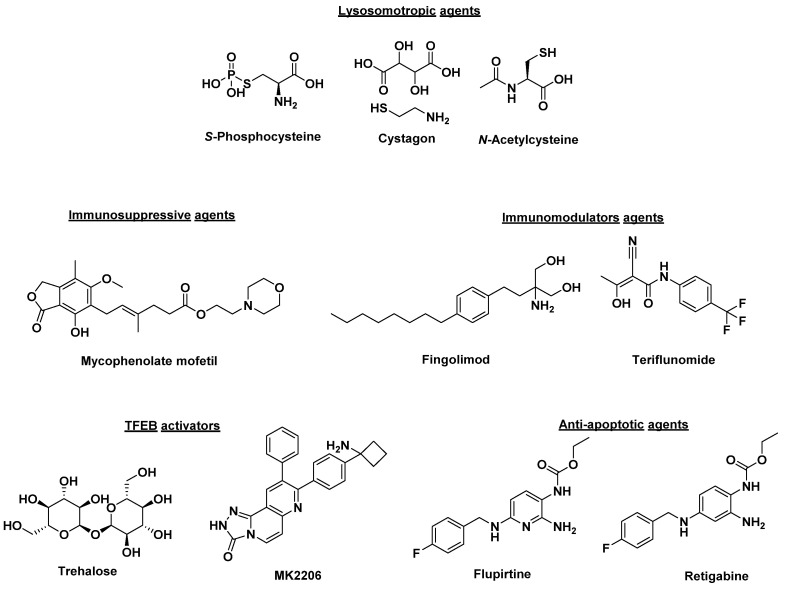
Structures of small molecules being investigated as potential NCL therapy.

Small molecule therapies that improve lysosomal or autophagic function and others that serve as immune modulators or neuroprotective agents have been extensively investigated (Figure 1) [44,45]. Lysosomotropic agents that include cysteamine, phosphocysteamine, cysteamine bitartrate (Cystagon) and *N*-acetylcysteine block ceroid accumulation by being transported to lysosomes and inhibiting the formation of cysteine thioesters [46,47]. Phosphocysteamine (Figure 1) reduced ceroid accumulation and storage materials in a CLN1 disease mouse model and CLN1 diseased patients [46,48,49]. Further, a pilot clinical trial (NCT00028262) using the combination of cysteamine bitartrate (Cystagon^®^) and *N*-acetylcysteine (Figure 1) resulted in a modest improvement in patients carrying severe CLN1 disease mutations [50]. Another class of therapeutics tested in a mouse model of JNCL are phosphodiesterase-4 (PDE4) inhibitors (such as rolipram, roflumilast, and PF-06266047) which showed an increase in cyclic adenosine monophosphate (cAMP) levels in *CLN3*^Δ*ex7/8*^ knockout mice with beneficial neurological effect [51]. *CLN3*^Δ*ex7/8*^ mice treated with PDE4 inhibitors showed increased brain cAMP levels and reduced neuronal apoptosis and neuroinflammation, and increased glutamate transporter expression [51]. Moreover, significant improvements in motor functions, decreased neuroinflammation, glial activation, and lysosomal pathology were observed compared to wild-type animals [51]. The immunomodulatory agents, fingolimod and teriflunomide (Figure 1), showed improved motor skills and reduced CLN3 disease severity when tested in a CLN3 disease mouse model [52]. Similarly, immunosuppressive agents such as mycophenolate mofetil demonstrated similar results when tested in a CLN3 disease mouse model [53]. In a double-blind placebo-controlled phase II trial (NCT01399047), mycophenolate mofetil was shown as well-tolerated in controlling secondary autoimmunity and neuroinflammation. However, it did not demonstrate definite effects to confer a clinical benefit [54]. Trehalose, a disaccharide composed of two glucose molecules, and MK2206, an anticancer agent, act by activating transcription-factor EB (TFEB) by inhibiting the serine/threonine kinase Akt, independently of the mechanistic target of rapamycin complex 1 (mTORC1), a known TFEB inhibitor [55,56,57]. This mechanism was shown to reduce lipofuscin buildup in CLN3 fibroblasts and mouse models [58]. One disadvantage of the translation of trehalose to the clinic is that the enzyme trehalase inactivates the small molecule through lysis in the small intestine. Pre-clinical evaluation of the intravenous delivery of trehalose, combined with miglustat, a trehalase inhibitor, is currently ongoing [59]. Other drugs with anti-apoptotic action in the NCLs, including flupirtine and retigabine, have shown promising results in inhibiting apoptosis, decreasing ceramide levels, and preserving cell survival in CLN1, CLN2, CLN3, CLN6, and CLN8 cultured patient lymphoblasts and neurons [44,60,61]. The study of flupirtine derivatives that enhance autophagy and induce the anti-apoptotic protein Bcl-2 in “neuron-like” PC12 cells and healthy iPSC-derived neurons has identified more potent analogs [62]. These potent compounds have resulted in the clearance of subunit c accumulation by mTOR-independent modulation of autophagy, conferred protective effects through the induction of Bcl-2, and rescued mitochondrial dysfunction in an iPSC-derived CLN3 neuron model [63]. Interestingly, in a CLN3 disease mouse model, results showed that different proteins were being impacted based on gender. Female mice treated with flupirtine showed a significant increase in Bcl-2 expression while male mice showed upregulation of Bcl-xl, an anti-apoptotic protein. Despite different proteins being affected in male versus female mice, this ultimately led to the upregulation of anti-apoptotic pathways and caused decreased cell death in the brains of flupirtine treated CLN3 mouse models [64]. 

## 3. NCL Animal Models

The use of animal models to study the NCLs has expanded in the last decade [65,66,67,68]. Animals that have naturally occurring neurological deficits relevant to the NCLs or are genetically engineered to mimic specific NCL genes were used as screening tools for evaluating potential therapies for human NCLs. These animals include large animals, such as dogs, pigs and sheep, or small rodents such as mice and rats, or non-mammalian models that include zebrafish, yeast, and fruit fly. Although each of these models have limitations and lack reliability regarding their translation to the clinic, they serve as tools to improve the understanding of the pathology of NCL disease progression, improve the accuracy of models of the NCLs and allow elucidation of therapeutic delivery mechanisms, pharmacodynamics, and toxicology. Genetically creating a homozygous deletion of either the entire exon 4 (*Ppt1*^Δ*ex4*^) or exon 9 (*Ppt1*^Δ*ex9*^) of the *PPT1* genome has replicated CLN1 disease phenotypic features, which includes the accumulation of autofluorescent granular osmiophilic deposits and prominent loss of GABAergic interneurons in several brain areas [69]. Pre-clinical evaluation of enzyme replacement and gene therapies were first performed in TPP1 deficient mice (*Tpp1^−/−^*), a model of CLN2 disease [70,71]. For CLN3 disease, several large gene deletion methods (*CLN3*^−/−^, *CLN3*^Δ*ex1−6*^, *CLN3*^Δex*7−8*^) have led to models that mimic the human mutation and convey similar disease progression that includes early glial activation followed by neuronal loss [72]. Anti-inflammatory and candidate gene therapy studies have used these mouse models for pre-clinical development [41,73,74]. Established animal models for studying retinal degeneration in CLN3 disease are lacking. Wiley and colleagues investigated the use of AAV-based gene augmentation to treat degenerative retinal blindness caused by CLN3 disease mutations. First, induced pluripotent stem cells (iPSCs) were generated from two patients harboring CLN3 mutations. A clinical-grade AAV2 carrying the full-length human *CLN3* gene was developed and delivered to patient-specific fibroblasts and iPSC-derived retinal neuronal cells in which it was able to restore the full-length CLN3 transcript and protein successfully. In follow-up in vivo toxicity studies in wild-type mice, the purified AAV2-*CLN3* showed no retinal toxicity [74]. This study provides meaningful insights and can potentially be the first step towards testing AAV-based gene augmentation clinical trials in children with CLN3 disease. To this end, mouse models are a vital part of NCL disease research; however, they face challenges for translation into the clinic, which include neurodevelopmental, neuroanatomical, and mechanistic differences between mice and humans and apparent phenotypic presentation of NCL models [75,76]. These differences hindered the success of several clinical trials wherein therapies with promising effects in mouse models lacked efficacy in patients [50,77].

## 4. NCL Cellular Models

Despite extensive research to understand the molecular cell biology of processes that lead to the neurodegeneration seen across the NCLs, the lack of phenotypic in vitro model systems for drug screening hinders clinical development. This leads to highly challenging drug discovery campaigns and limits the information about natural disease history and its incompletely characterized pathophysiology. The recently reviewed cellular models of Batten disease by Minnis et al. carry many advantages in elucidating complex biological systems [78]. However, they carry fundamental limitations that hinder their use as drug screening models. Yeast models offer a unique experimental model system to examine protein functions or cellular pathways that mediate the secretion, aggregation, and subsequent toxicity of proteins associated with mutations in CLN genes [79]. However, yeast is primitive when compared to neurons, and their natural unicellular classification lacks morphological structures such as dendrites, axons, synapses, and associated specialized functions [80]. *Dictyostelium discoideum* is an amoeba proven to be an excellent model organism to research neurodegenerative diseases, including the NCLs [81]. However, the model has several disadvantages including that glycosylation pattern may differ from human native protein, preferential codon usage, and cultivation processes have yet to be optimized for industrial scale production [82]. Mouse cellular models are also used in studying the NCLs [65] but carry the known disadvantage that mouse brain cells have distinct biological functions compared to those of humans. It can also be argued that predictions of drug efficacy in human neurodegenerative diseases using mouse models are mostly unsuccessful [83,84]. Human primary fibroblasts and lymphoblasts are widely utilized and were historically included for biochemical confirmation of disease. However, using these models can be unreliable as the pathology of Batten disease is incomplete in the skin or blood [85,86]. Exploring new technologies to generate in vitro models from patient-derived iPSCs has gained increasing traction, especially in neurodegenerative diseases [87,88,89,90].

## 5. iPSC-Based Phenotypic Modeling for Drug Discovery in The NCLs

Recent advances in the ability to reprogram patient iPSCs have provided a novel means to generate phenotypic disease-relevant cells for in vitro disease modeling [91,92]. Technology introduced by Yamanaka and colleagues using the transcription factors OCT4, SOX2, KLF4, and c-MYC in somatic cells to generate human iPSCs established a reliable method to produce stem cells [92,93]. Practically, human iPSCs can differentiate into any cell type of the human body; thus, patient iPSCs can provide a source of cells that foster a precise pattern of genetic variants with the advantage of acquiring the pathogenesis of the disease and recapitulating phenotypes of various human neurodegenerative diseases in an appropriate microenvironment. Consequently, iPSCs have become well-established models for some human diseases [94,95,96]. In addition, advancements in techniques used in iPSC culture and the development of robust patient cell maturation and differentiation protocols have enabled plausible methods for phenotypic drug screening in iPSC-derived disease-target cells [93,97,98]. However, several technical aspects should be considered when applying this approach. For instance, variability in the phenotypes of iPSC lines derived from individual patients can result in misleading pathological mechanisms or drug effects. Therefore, current gene-editing technology, such as clustered regularly interspaced short palindromic repeats (CRISPR), has allowed researchers to regulate genetic background by generating isogenic control lines for a specific patient sample and a set of genetically defined human iPSC lines for disease modeling [99,100]. For example, Zhang et al. generated a human iPSC line (LEIi004-A) from a patient diagnosed with late-onset non-syndromic CLN3-associated retinitis pigmentosa [101]. Primary dermal fibroblasts were reprogrammed using non-viral and non-integrating episomal plasmids expressing OCT4, SOX2, KLF4, L-MYC, LIN28, p53 short hairpin RNA, and mir302/367 microRNA. Additionally, the same group created an isogenic control line (LEIi004-A-1) using CRISPR/Cas9 gene editing to correct a *CLN3* mutation. Burnight et al. also used CRISPR/Cas9 gene editing to successfully correct genomic DNA and restore the *CLN3* transcript in two independent patient iPSC lines [102]. Their studies revealed that correction of the genomic deletion was substantially more difficult in the patient-derived iPSCs obtained from a heterozygote individual compared to an individual being homozygous for the common 1.02 kb deletion. In another model of CLN3 disease, CRISPR/Cas9 gene-editing technology was also used to introduce a disease-causing mutation into healthy human iPSCs in a cerebral organoid model [103]. The model was used to study early brain development; results obtained from this study revealed that a mutation in *CLN3* severely affected brain development as some of the CLN3 mutant organoids were unable to develop normally while the rest demonstrated transcriptional and metabolomic changes, which in turn affected development, corticogenesis, and synapses. Decreased cerebral tissue metabolites such as creatinine and gamma-aminobutyric acid (GABA) were also observed. These results suggest that a mutation in CLN3 disease can cause neurodevelopmental changes. Overall, these gene-corrected, patient-derived iPSCs provide essential insights towards the treatment of Batten disease. Another hurdle for modeling disease with iPSC-derived cells is that the maturity of derived neurons and differentiation time required for phenotypes to emerge may vary across iPSC lines [104]. 

Given the great potential for the use of iPSC technology in developing a phenotypic model for human disease [99,100], we provide an overview of iPSC technology in modeling NCL diseases in the following section, including essential findings in drug development and newly established iPSC models that can be used for drug screening in NCL diseases (Table 2).

Sima et al. differentiated neural stem cells (NSCs) from three patient dermal fibroblast lines (purchased from the Coriell Cell Repository): one from a CLN1 patient carrying a mutation in *PPT1* gene that encodes the enzyme PPT1, and two from CLN2 patients carrying mutations in the *TPP1* gene that encodes the enzyme tripeptidyl peptidase 1. The deficiency in lysosomal enzymes PPT1 in CLN1 disease or TPP1 in CLN2 disease results in lysosomal accumulation of lipids and subsequently the enlargement of lysosomes in patient cells, lipid droplet accumulation, and lysosomal storage of subunit c [105]. The differentiation of iPSCs derived from those patients to NSCs has afforded a valid disease model that can be used in the phenotypic screening of drugs that ameliorate disease state. For instance, enzyme replacement therapy rescued lipid accumulation and lysosomal enlargement in the diseased fibroblast cells. This study identified that δ-tocopherol (DT) was able to rescue the NCL phenotypes of enlarged lysosomes with a reduction in lipid droplet accumulation and lysosomal storage of subunit c by approximately 40% [105].

Lojewski et al. generated iPSCs from two CLN2 and four CLN3 disease patients with mutations in *TPP1* and *CLN3*, respectively [106]. The developed iPSCs possessed abnormal lysosomal and autophagic compartments. The differentiation of CLN3 patient-derived iPSCs to neural progenitor cells (NPCs) showed distinct Golgi and endosomal/lysosomal marker staining patterns compared to control and exhibited NCL-like storage and organelle abnormalities. To validate this model as a screening model, the overexpression of non-mutated *TPP1* or *CLN3* was able to rescue subunit c accumulation in lysosomes in the patient-derived NPCs [106]. These findings validated AAV-mediated gene therapy, currently in clinical trial (NCT01414985).

In a follow-up study, the derivation of iPSCs from CLN3 disease patients and subsequent differentiation to NPCs were used as a phenotypic model to validate the effect of a small molecule autophagy modifier, identified from a screen conducted in a homozygous Cb*Cln3*^Δ*ex7/8*^ cell line stably expressing GFP-LC3 transgene [109]. This model demonstrated the accumulation of the autophagy protein LC3 by constitutively expressing a GFP-LC3 fusion. A novel biochemical assay was developed as a screening tool to validate drug efficacy. Thapsigargin, an ER Ca^2+^ ATPase inhibitor, was found to increase GFP-LC3 puncta in *CLN3^Ivs13/E15^* and *CLN3^hom^*^Δ*ex7/8*^ iPSC-derived NPCs. The study linked CLN3 disease mutation to abnormal maintenance of intracellular Ca^2+^ storage, supporting findings that Ca^2+^ and other ions induce homeostatic disturbances present in other forms of NCL [110].

In another engineered model for drug screening of CLN3 disease, Kinarivala et al. demonstrated the characterization of a dysfunctional CLN3 patient iPSC-blood-brain barrier (BBB) and detailed a CLN3 diseased patient iPSC-derived neuron model that recapitulates multiple phenotypes of the disease [63]. This study was the first to report an iPSC-derived model of the BBB for CLN3 disease. Brain microvascular endothelial cells (BMECs) differentiated from CLN3 patient-derived iPSCs showed an impaired barrier phenotype, a compromised barrier function, and an angiogenic phenotype compared to the control. The developed neural model demonstrated the inclusion of lipofuscin within lysosomes and the accumulation of subunit c of ATP synthase, which implicated dysfunctional autophagy. Furthermore, the iPSC model showed mitochondrial impairment, the alteration of lysosomal pH and accelerated apoptosis of neurons, recapitulating key phenotypes of CLN3 disease. This phenotypic model may be suitable for screening drugs that act as autophagy modulators, anti-apoptotic compounds, and compounds with effects on mitochondria, or a combination. The model was used to screen constituent compounds of an in-house library which resulted in upregulated expression of Bcl-2, suppressed ceramide levels and activation of autophagy, directly countering three phenotypes of CLN3 disease. Furthermore, subunit c of mitochondrial ATP synthase accumulation was significantly reduced upon treatment with selected compounds. This model was the first reported to employ CLN3 patient iPSC-derived neurons for small molecule screening and reasoned that developing a phenotypic assay around autophagy in an accurate genetic model could serve as a useful starting point in fulfilling the promise of iPSC technology to discover new therapeutics for the NCLs. 

More recently, a study aimed to understand the role of CLN3 in retinal pigment epithelium (RPE) structure and function by generating iPSC-RPE cells from fibroblasts of CLN3 disease patients has implicated the contribution of the *CLN3* gene in RPE dysfunction and photoreceptor outer segment (POS) phagocytosis in CLN3 disease [107]. The CLN3 iPSC-RPE cells showed an affected structure and function in a cell autonomous manner when compared to control; moreover, the RPE structure necessary for POS phagocytosis was also abnormal and decreased apical RPE microvilli density, and reduced POS binding was also observed which resulted in the lower uptake of POS by CLN3 iPSC-RPE cells. Consistent with the impaired lysosomal degradation of POS by RPE cells in CLN3 disease pathology [111], CLN3 in iPSC-RPE cells was colocalized with the lysosomal marker LAMP1. This study, therefore, suggested that RPE cells may be a crucial gene therapy target to combat the loss of vision phenotype in CLN3 disease [107].

Another study focused on developing a CLN5 disease model that demonstrates phenotypic characteristics has employed CLN5 patient-derived iPSCs. Upon differentiation into neurons, these cells exhibit the accumulation of autofluorescent storage material and subunit c of mitochondrial ATP synthase. Further, morphological changes in the intracellular organelles of both the lysosomal compartment and the ER were detected. These findings reiterate previous findings of disturbances in lysosomal structures and ER stress in CLN5-deficient cells. Most of the disease mutations lead to the retention of the protein to the lysosome and ER [108]. 

While only a limited number of NCL iPSCs are currently described in the literature, efforts continue apace to provide more models for the research community. The New York Stem Cell Foundation (NYSCF) has partnered with the Beyond Batten Disease Foundation (BBDF) to create a collection of CLN3 iPSCs from 24 individual patients and family members to be made commercially available. Similarly, the iPSC core at Cedars Sinai has recently made available to the research community a number of CLN6 iPSC lines.

## 6. Conclusions

The NCLs encompasses a group of rare, fatal, pediatric neurodegenerative lysosomal storage disorders. Several gene mutations (CLN1–CLN8, CLN10–CLN14) can lead to NCL; however, a partial understanding of the function of the disease-associated proteins has hindered therapy development. Current treatment options are only symptomatic and focus on delaying progression. To date, there are only two clinically approved drugs, Brineuria, for the treatment of CLN2 disease, and Neurogene’s recently approved gene therapy to treat CLN5 disease. Different organism models have become available for NCL disease research which have provided a myriad of important information about the protein function or dysfunction for each of the associated genes, possible disease mechanisms, and have enabled detailed preclinical studies and in a small number of cases, clinical trials. 

Herein, we have highlighted the contributions of different disease models to NCL research, focusing on the established patient-derived iPSC phenotypic screening models. The ability of iPSCs to encompass the precise pattern of genetic variants, along with acquiring disease pathogenesis and phenotype makes them a more translational model compared to mice and eliminates the problem of species difference. However, compared to animal models, fewer iPSC models currently exist. 

The brain is a complex network of many different cellular phenotypes and screening compounds in just one phenotype, i.e., neurons, is not a complete representation of the environment in the brain. While most studies in NCL patient-derived iPSCs employ either NPCs or neurons there are emerging studies looking at biochemical and pathophysiology effects of NCL on other cell phenotypes, one such example is the use of BMECs to model the blood-brain barrier that identified an impaired barrier phenotype in CLN3. Differentiation of iPSCs into other phenotypes including oligodendrocytes, astrocytes, microglia etc. is ongoing and results are expected in due course. These cell types will allow the construction of increasingly complex co-culture models that more readily represent the human brain and thus allow a greater understanding of the disease. However, for drug screening processes, these co-culture systems are complex, expensive, and unsuitable for high throughput. Thus, at the present time, phenotypic screening of compound libraries will likely continue to focus on one cellular type with NPCs representing an excellent phenotypic model combined with relatively low cost and rapid growth and characterization. Once promising compounds are identified, further studies in other cell types will be warranted to fully characterize the effects of a lead compound.

Despite this scarcity of NCL patient iPSCs available to the research community, the past years saw reports of the first iPSC-based screenings to identify new hit and lead compounds for NCL drug discovery. The increasing commercial availability of CLN3 patient-derived iPSC cell lines opens this highly phenotypic resource up to the non-specialized researcher. Thus, the drug discovery specialist has a new tool to enhance translational therapeutic discovery for the NCLs, through which the identification and development of novel therapeutic options to help improve lifespan and quality of life for children suffering from this deadly disease may be facilitated.

## Figures and Tables

**Table 1:** NCL Diseases and Encoded Genes/Proteins [13,14,15].

Disease	GENE/Protein	Age of Onset	Known Function	Refs.
**CLN1**	*PPT1* (palmitoyl protein thioesterase 1)	6–18 months	Palmitoy-protein thioesterase activity plays a critical role in the degradation of lipid-modified proteins via removing fatty acid residues from cysteine residues	[16]
**CLN2**	*TPP1* (tripeptidyl peptidase 1)	2–4 years	Serine protease activity prevents intralysosomal accumulation of storage material and neuronal loss	[17,18]
**CLN3**	*CLN3*, lysosomal/endosomal transmembrane protein	4–10 years	Predicted function as a pH regulator and modulator of vesicular trafficking and fusion that promotes cellular homeostasis and neuronal survival	[19]
**CLN4**	DNAJC5/CSPα (cysteine string protein α)	Adult	Involvement in exocytosis and endocytosis functions plays a regulatory role in ATPase activity and assists in folding proteins in synaptic vesicles	[20]
**CLN5**	Soluble lysosomal protein	4–7 years	Glycoside hydrolase activity modulates vesicular trafficking	[21,22]
**CLN6**	Transmembrane protein of endoplasmic reticulum	18 months to 6 years	Precise function remains unclear but is linked with intracellular trafficking and lysosomal function	[23]
**CLN7**	MFSD8 (major facilitator superfamily domain-containing 8), lysosomal transmembrane protein	2–6 years	Predicted transmembrane transporter function plays a role in preventing neuronal loss, robust accumulation of lipofuscin, reactive gliosis, and degeneration and storage accumulation in the retina	[24]
**CLN8**	Transmembrane protein of endoplasmic reticulum	2–7 years (Turkish variant late-infantile NCL), 5–10 (northern epilepsy)	Aids in lysosomal biogenesis through transportation from the ER to the Golgi complex and in the regulation of lipid metabolism	[25,26]
**CLN10**	CTSD (cathepsin D)	In utero	Aspartic protease functions in an unknown neuroprotective mechanism	[27,28]
**CLN11**	PRGN (progranulin)	Early to mid-twenties	Known roles in inflammation, embryogenesis, cell motility and tumorigenesis	[29]
**CLN12**	ATP13A2	13–16 years	Regulation of ion homeostasis	[30]
**CLN13**	CTSF (cathepsin F)	Adult	Loss of lysosomal cysteine protease activity leads to deterioration of motor function and reduced brain function	[31]
**CLN14**	KCTD7 (potassium channel tetramerization domain-containing protein 7)	8–24 months	Modulation of potassium ion channel activity	[32,33]

**Table 2:** Currently available NCL iPSC lines.

Name	NCL	Controls	Treatment	Summary	Refs.
New York Stem Cell Foundation (Multiple)	CLN3	Parent cells available	*-*	-	https://nyscf.org/
Cedars Sinai iPSC Core (Multiple)	CLN6	Parent cells available	-	-	https://biomanufacturing.cedars-sinai.org/
LEli004-A	CLN3	Isogenic (LEli004-A-1)	-	-	[101]
Sima et al.	CLN1 & CLN2	WT control	δ-Tocopherol (DT) and hydroxypropyl-β-cyclodextrin (HPBCD)	Treatment reduced lipid accumulation and lysosomal enlargement	[105]
Lojewski et al.	CLN2 & CLN3	WT control	Fenofibrate, gemfibrozil and PTC124	Fenofibrate and gemfibrozil failed to increase TPP1 activity. While PTC124 resulted in an increase of TPP1 activity and attenuation of neuropathology in patient iPSC-derived neural progenitor cells	[106]
Wiley et al.	CLN3	IMR90 control	Adeno-associated adenovirus serotype 2 (AAV2) carrying human CLN3	AAV2-CLN3 restored CLN3 patient-specific transcript and protein in fibroblasts and iPSC-derived retinal neurons	[74]
Kinarivala et al.	CLN3	IMR90 control	Flupirtine derivatives	Neuroprotective molecules upregulated Bcl-2, modulatedautophagy, enhanced clearance of subunit c and rescued mitochondrial dysfunction	[63]
Tang et al.	CLN3	WT control	*CLN3* gene supplementation	Gene therapy rescued phagocytosis of photoreceptor outer segment in CLN3 disease iPSC-RPE cells	[107]
Uusi-Rauva et al.	CLN5	WT control	-	Phenotypic characterization of CLN5 patient-derived iPSCs showed accumulation of autofluorescent storage material and subunit c of the mitochondrial ATP synthase	[108]
